# Mesenchymal Stromal Cells Can Regulate the Immune Response in the Tumor Microenvironment

**DOI:** 10.3390/vaccines4040041

**Published:** 2016-11-08

**Authors:** Alessandro Poggi, Massimo Giuliani

**Affiliations:** 1 Molecular Oncology and Angiogenesis Unit, IRCCS AOU San Martino IST, 16132 Genoa, Italy; 2 Laboratory of Experimental Cancer Research, Department of Oncology, Luxembourg Institute of Health, Luxembourg City L-1526, Luxembourg; giulianimas@hotmail.com or massimo.giuliani@lih.lu

**Keywords:** mesenchymal stromal cells, NKG2D-NKG2DL, innate immunity, γδ T cells, immune regulation, hematopoietic malignancies, immune escape, NK cells

## Abstract

The tumor microenvironment is a good target for therapy in solid tumors and hematological malignancies. Indeed, solid tumor cells’ growth and expansion can influence neighboring cells’ behavior, leading to a modulation of mesenchymal stromal cell (MSC) activities and remodeling of extracellular matrix components. This leads to an altered microenvironment, where reparative mechanisms, in the presence of sub-acute inflammation, are not able to reconstitute healthy tissue. Carcinoma cells can undergo epithelial mesenchymal transition (EMT), a key step to generate metastasis; these mesenchymal-like cells display the functional behavior of MSC. Furthermore, MSC can support the survival and growth of leukemic cells within bone marrow participating in the leukemic cell niche. Notably, MSC can inhibit the anti-tumor immune response through either carcinoma-associated fibroblasts or bone marrow stromal cells. Experimental data have indicated their relevance in regulating cytolytic effector lymphocytes of the innate and adaptive arms of the immune system. Herein, we will discuss some of the evidence in hematological malignancies and solid tumors. In particular, we will focus our attention on the means by which it is conceivable to inhibit MSC-mediated immune suppression and trigger anti-tumor innate immunity.

## 1. Introduction

Within the tumor microenvironment (TME), several cell populations can impair immune response and thus these cells can be considered as additional targets for therapy in hematological malignancies and solid tumors such as carcinomas [1,2]. Mesenchymal stromal cells (MSC) such as fibroblasts (cancer-associated fibroblasts(CAF) or tumor-associated fibroblasts (TAF)), subsets of inflammatory innate immune cells, fibrocytes, pericytes, blood, and lymphatic vessels are all players in immune regulation [1,2]. The tumor niche is a place where tumor cells try to expand to overcome all the cellular and molecular mechanisms set up by the host to limit tumor overgrowth. Of course, tumor cells are ill while the TME is mainly composed of healthy MSC that respond to tumor noxa trying to repair the tissue. Indeed, some subsets of MSC can differentiate into specialized mesenchymal cells that are essential to tissue healing [2,3]. However, the repair is not possible unless tumor staminal cells are eliminated by therapy or the immune system [2]. Thus, to favor its own growth, the tumor modulates its behavior and immunological status, “educating” the TME [2,3]. It is conceivable that TME-targeted strategies would be very attractive to trigger an integrated cancer therapy. MSC can influence TME to produce extracellular matrix (ECM) and soluble factors involved in survival and proliferation of stem cells in hematological malignancies and solid tumors, as well as in epithelial mesenchymal transition (EMT) and metastasis of carcinoma cells [1,4]. In addition, MSC can favor tumor cell growth, inhibiting both innate and adaptive immune cell response [5,6]. Some reviews recently published have focused their attention on MSC as regulators of immunity [2,3]. Herein, we will describe MSC phenotypic and functional features, focusing on their regulatory activities on innate anti-tumor effector lymphocytes as natural killer (NK) CD3^−^CD56^+^ and γδ T cells. Furthermore, we will analyze the feasibility of using drugs targeted to MSC and whether it is conceivable to imagine MSC as a vaccine tool to transform TME into an inadequate niche for the survival and proliferation of tumor cells.

## 2. MSC Phenotypic and Functional Features Relevant in the Interaction with Tumor and Immune Cells

### 2.1. MSC Phenotypic Characterization and Function: Cell Membrane-Expressed Enzymes

Since MSC represent a quite heterogeneous cell population present in TME, a specific marker is still elusive [2,3]. MSC are responsible for the production and secretion of several components of ECM and the molecular and functional characteristics of ECM are greatly influenced by tumor cells [2,3]. MSC express enzymes that are responsible for the synthesis of ECM as proly-4-hydroxilase involved in the hydroxylation of collagen [2]. Also, the expression of fibroblast activation protein (FAP), a member of the serine protease family, is a characteristic of some fetal mesenchymal tissues and TAF [7,8,9,10,11]. FAP is apparently little expressed or even absent in adult and healthy fibroblasts but can be re-expressed during wound healing, osteo- and rheumatoid arthritis, pulmonary fibrosis, and cirrhosis [11]. Importantly, the proteolytic activity of FAP can support tumor growth and proliferation. Indeed, FAP has both dipeptidyl peptidase and endopeptidase activity that can determine the collagen I and gelatin degradation [11]. Thus, FAP can remodel the ECM and influence tumor cells’ growth by promoting angiogenesis [11]. However, it has been reported that FAP can also function as a tumor suppressor [11]. These findings would suggest that FAP can show different functional behavior depending on the surrounding microenvironment [11,12]. Furthermore, FAP is expressed on several tumor cells and thus it is difficult to define the relative contribution of MSC and neoplastic cells to TME due to this enzyme [11,12]. CD73, an ecto-5’-nucleotidase is a good marker to characterize MSC but it is widely expressed on cancer cells, lymphocytes, and accessory cells [13,14,15]. CD73 is active as a disulfide-linked homodimer; this enzyme catalyzes the hydrolysis of the extracellular adenosine monophosphate (AMP) to adenosine [13,14,15]. Indeed, adenosine can bind to distinct G-protein-coupled receptors, influencing the activity of adenylyl cyclase, cyclic AMP production and protein kinase A (PKA) activation. Interestingly, CD73 is expressed not only on MSC but also on tumor cells and Treg cells, suggesting that the adenosine found within TME can be derived from different sources, to further amplify the strong inhibitory effect derived from a single cell type [13,14,15]. Transglutaminase (TG)-II can be used to selectively identify MSC in immunohistochemistry and cytofluorimetry [16,17,18,19,20,21,22,23]; however, it is also expressed on tumor cells undergoing EMT. TG-II is involved in several processes that can regulate tumor cell proliferation and apoptosis [16,17,18,19,20,21]. MSC also express matrix metalloprotease (MMP) and some members of a disintegrin and metalloproteases (ADAM) family [24,25,26]. These enzymes are involved in cell development, migration, and wound healing [27,28,29,30,31]. Some of their substrates are growth factors or cytokines such as TNF-α and EGF, relevant for development and tumor cell expansion [27,28,29,30,31]. Furthermore, MMP and ADAMs can regulate the shedding from both MSC and tumor cells of ligands for activating receptors expressed on effector lymphocytes [2,3]; this mechanism leads to the impairment of tumor cell recognition, thus indicating that MSC can inhibit tumor cell elimination acting on immune cells [2,3]. These findings indicate that MSC can regulate the TME microenvironment with several surface-expressed enzymes impairing lymphocyte recognition and influencing tumor cell proliferation and apoptosis.

### 2.2. Surface Molecules Expressed on MSC Involved in the Interaction with the Immune System

MSC express some counter ligands of lymphocyte receptors that have been extensively reviewed previously [2]. Briefly, the intercellular cell adhesion molecule (ICAM)-1 and the lymphocyte function-associated antigen (LFA)-3, two ligands of LFA-1 and CD2 lymphocyte counter receptors, respectively, can be expressed on MSC and they are involved in the lymphocyte-MSC interaction [2]. In addition, it has been shown that cytolytic effector cells can recognize MSC through the natural-killer group 2 member D (NKG2D), which interacts with NKG2D ligands (NKG2DL), namely UL16 binding proteins such as ULBP3 and MHC class I polypeptide-related sequence A/B (MICA/B) expressed on MSC [24,25,26,32,33,34].

Most importantly, these NKG2DL expressed on MSC can be shed in the extracellular milieu, thus interfering with NKG2D-mediated recognition of tumor target cells [24,25,26]. Indeed, ADAMs can function as sheddases of NKG2DL [35], contributing to the immune cell escape of neoplastic cells both in carcinomas and hematological malignancies [32,33,34,35]. MSC can bear ligands for surface molecules involved either in the activation or inhibition of lymphocyte functions [2,3]. Indeed, DNAX accessory molecule 1 (DNAM-1) can react with MSC-expressing polio virus receptor (PVR, CD155) and nectin 2 (CD112),which are two DNAM-1Ls [36,37]. This recognition should usually lead to killing of the DNAM-1L-bearing cell [36,37]. However, it is also possible that DNAM-1L can bind T cell immunoglobulin and ITIM domain (TIGIT) or CD96 inhibitory molecules on lymphocytes, leading to the impairment of immune response [38]. Moreover, NKG2DL and DNAM-1L are widely expressed in several carcinomas and leukemic disorders, suggesting that MSC, rather than tumor cells, can be an suitable alternative target in TME [36,37]. It is of note that NKG2D and DNAM-1 are expressed not only on NK cells and γδ T cells but also on CD8^+^ antigen-specific αβ T cells, suggesting that the cytolytic activity of these latter cell populations can also be diverted from tumor to MSC. Nonetheless, this specific effect is still to be clearly demonstrated in vivo. Among the activating receptors expressed on cytolytic effector cells, the Natural Cytotoxic Receptors (NCR) such as NKp30, NKp44, and NKp46 should also be considered [39]. In this case, their expression appears to be restricted to NK cells and unique subsets of γδ T cells [40]. However, the role of NCR in the cross-talk between MSC and effector lymphocytes is still to be defined. Most importantly, both NK and γδ T cells can express a plethora of inhibitory receptors for Human Leukocyte Class-I antigen (HLA-I), such as the Killer Inhibitory Receptors (KIR), and C-type lectin inhibitory receptors (CLIR) such as NKG2A [41]. Furthermore, some members of the inhibitory leukocyte immunoglobulin-like receptors family (LILRB) can downregulate NK and γδ T cells’ cytolysis [42]. MSC express HLA-I antigens and it is conceivable that the interaction of inhibitory receptors expressed on innate immune cells with HLA-I can downregulate the lymphocyte response [41]. Altogether, these findings indicate that MSC can regulate anti-tumor effector cells through the balance between positive and negative signals delivered during the MSC-lymphocyte cross-talk [2].

## 3. Molecular Mechanisms Responsible for the Immunosuppressive Effect Mediated by MSC in TME

**Figure 1:**
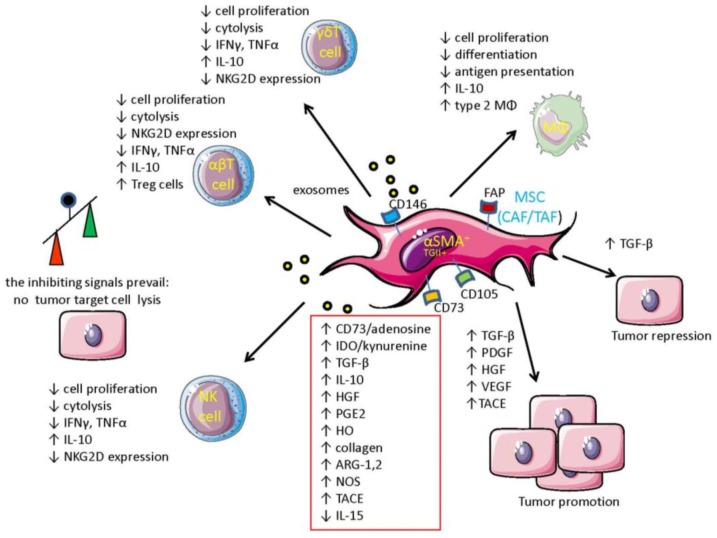
MSC-dependent molecular mechanisms mediating immune regulation in the tumor microenvironment and cross-talk with tumor cells. Mesenchymal stromal cells (MSC) such as cancer- or tumor-associated fibroblasts (CAF/TAF) can regulate the immune response in the tumor microenvironment (TME) by secreting a plethora of inhibitory factors (indicated in the red box). These factors can be released as soluble molecules or as a component of exosomes. These factors can inhibit cell proliferation, cytolysis, and production of anti-tumor lymphokines by effector anti-tumor lymphocytes such as natural killer (NK), αβ T, and γδ T lymphocytes. Furthermore, some of these inhibitory factor such as TGF-β can downregulate the expression of NKG2D-activating receptors, leading to the impairment of recognition and consequent killing of tumor cells expressing NKG2D-L. Overall, the inhibitory signals present in TME overcome positive signals (see the balance depicted on the left) favoring tumor cell growth. In addition, the generation of regulatory T cell (Treg) and myeloid-derived suppressor cells (MDSC) is promoted by MSC. Finally, MSC can trigger proliferation of tumor cell-secreting growth factors such as PDGF, HGF, and TGF-β. MSC, through the action of the tumor necrosis factor-α-converting enzyme (TACE), can release from the tumor cell surface amphiregulin, favoring tumor cell invasiveness. Also, VEGF produced by MSC can promote tumor tissue neovascularization. It is of note that TGF-β can function as a tumor suppressor rather than a tumor promoter.

MSC regulate immune response in several ways (Figure 1) [2,3]. Indeed, MSC downregulate effector lymphocyte activation using indoleamine 2,3 dioxygenase (IDO), heme oxygenase (HO), arginase 1 and 2 (ARG_1-2_), nitric oxidase synthase 2 (NOS_2_), hepatocyte growth factor (HGF), TGF-β, IL10, and prostaglandin E2 (PGE_2_) [43,44,45,46,47,48]. Some of these inhibitory molecular mechanisms, such as IDO, PGE_2_, TGF-β, and IL-10, are shared with myeloid-derived suppressor cells, tumor cells, and infiltrating Treg lymphocytes present in TME [49,50]. IDO and PGE_2_ are usually upregulated upon contact with inflammatory stimuli such as IFN-γ; thus, these mechanisms should be considered a way to switch off the inflammatory immune response to either exogenous or endogeneous danger signals. In this context, immunosuppression mediated by these factors should be considered a physiological response to damage and a drawback of a repair mechanism aimed to maintain tissue homeostasis [2,3,51]. Among the several inhibitory factors secreted by MSC, TGF-β is critical to trigger the generation of both conventional CD4^+^ Treg and γδ regulatory T cells [52,53]. This indicates that MSC can indeed skew the differentiation/maturation of several effector lymphocytes. In addition, TGF-β is a potent regulator of cytokine-induced upregulation of the NKG2D receptors on NK, CD8^+^ αβ T cells, and γδ T cells [2]. This effect can indeed impair the recognition of tumor cells expressing NKG2D-L and thus limit stress immunosurveillance [2]. PGE_2_ is certainly a key regulating factor secreted during the interaction between MSC and NK cells. Indeed, it has been reported that the IL-2-dependent upregulation of some NCRs and DNAM-1 expression is impaired on NK cells co-cultured with TAF isolated from melanoma lesions. This led to deficient NK cell-mediated lysis of melanoma target cells [54]. Also, arginase II (ARG_2_) expressed in α-smooth muscle actin (SMA)^+^ TAF [46] can convert arginine in ornithine, inhibiting tumor infiltrating lymphocyte (TIL) functions; this can happen in hypoxic conditions, impairing the elimination of tumor target cells. In addition, some reports support the idea that MSC can display heterogeneous behavior, facilitating the production of IFN-γ by effector lymphocytes [46]. Taken together, all these data would suggest that MSC present within TME are in different functional stages and the cytokine milieu can markedly influence their functional behavior and their immunosuppressive properties.

## 4. MSC Cross-Talk with Tumor Cells

In this paragraph, we will focus on the interaction between MSC and tumor cells from hematological neoplasia and briefly summarize some of the evidence dealing with cross-talk between MSC and solid tumor cells.

### 4.1. MSC–Tumor Cell Cross-Talk in Hematological Malignancies

**Figure 2:**
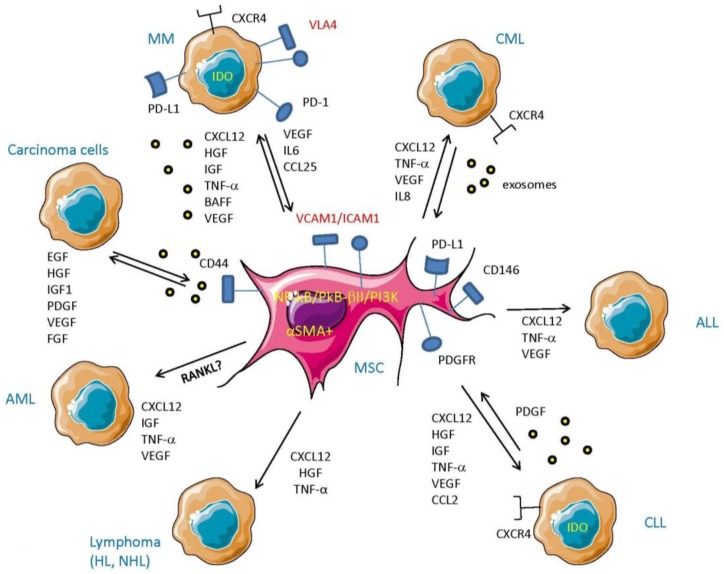
Cross-talk between MSC and tumor cells. MSC mainly through the production of CXCL12 (stromal derived factor-1), which, interacting with CXCR4, can influence the fate of several kinds of leukemic cells by triggering their proliferation, spread, and survival. Also, secretion as soluble or exosome-associated molecules of IL-8, insulin growth factor (IGF), hepatocyte growth factor (HGF), B-cell activating factor of the TNF family (BAFF), TNF-α, and vascular endothelial growth factor (VEGF) markedly influences the survival, expansion, and localization of leukemic and lymphoma cells. Platelet-derived growth factor (PDGF) is an example of the response of leukemic cells to trigger MSC and secrete in turn leukemic cell-sustaining cytokines. Some of the molecules that can be targeted to impair MSC–tumor cell interaction are shown to complete the scenario.

Bone marrow stromal cells (BMSC) are the major component of the hematopoietic stem cells (HSC) niche in the BM microenvironment [55,56]. They have the capacity of multi-lineage differentiation and display strong immunosuppressive properties, making them suitable for regenerative medicine and transplantation. However, BMSC are also important components of the TME and play a major role in tumor progression and immuno-escape by impairing the immune response and facilitating tumor evasion of immune cell-mediated surveillance [2,57,58,59,60]. Of note, non-activated stromal cells can be recruited to the tumor site and educated to differentiate into CAFs. By remodeling the ECM and producing growth factors, new and already differentiated CAFs can promote tumor growth, angiogenesis, and drug resistance [2,61,62,63,64]. Notably, BMSC can assume a CAF-like phenotype after prolonged exposure to tumor-conditioned media and can participate in tumor growth both in vitro and in vivo [62,64,65,66,67]. In addition, BMSC secrete several cytokines such as IL-6, IL-10, CCL5, and VEGF, which promote angiogenesis and the growth of cancer cells [2,58,60] (Figure 2). In the last decade it has been shown that the stromal microenvironment is a critical player not only in solid tumor survival and expansion, but also in hematopoietic malignancies pathogenesis [68,69,70,71]. Although the mechanisms that underlie the cross-talk between BMSC and hematopoietic cells need further investigation, several groups have demonstrated that stromal cells support tumor cell growth and immune escape.

#### 4.1.1. MSC in Chronic Lymphocytic Leukemia (CLL) and Chronic Myeloid Leukemia (CML)

Advanced evidence supports the finding that CLL cells are attracted to stromal cells, which protect them from apoptosis [72]. Interestingly, supernatants derived from CLL cells induce the PDGF receptor expression on stromal cells, whereas the interaction of BMSC and CLL cells promotes protein kinase C (PKC)-II and NF-κB activation in BMSC [72,73]. In addition, soluble factors secreted by stromal cells isolated from CLL patients contain high amounts of CXCL12 (SDF-1), which contributes to the decreased expression of CXCR4 in CLL cells and their infiltration in the bone marrow [74]. Additionally, Paggetti and colleagues have recently demonstrated that exosomes, small heterogeneous membrane vesicles of 40–100 nm involved in cell-to-cell communication, play a key role in the CLL-mediated differentiation of stromal cells to CAF-like cells [67]. These latter cells then contribute to the tumor progression by migrating and secreting soluble factors. A similar mechanism has been shown in CML. Indeed, exosomes isolated from CML cells promote IL-8 secretion by stromal cell [75]. IL-8 secretion thus induces CML cells’ survival, adhesion, and migration.

#### 4.1.2. MSC in Acute Myeloid Leukemia (AML)

Although most stromal cells isolated from AML patients displayed a classic MSC phenotype [55], recent evidence has shown that AML stromal cells also express high levels of CD146 compared to healthy donors [76]. Interestingly, Huang and colleagues reported that in AML patients stromal cells exhibit aberrant cytogenetics and cytokine pattern [77]. Furthermore, recent results suggest that AML stromal cells have a reduced ability to support hematopoietic differentiation [78]. In addition, CXCR4 and CD44 expressed on AML cells participate in the drug resistance induced by stromal cells [79,80,81,82,83,84]. For an overview of the therapy to target the interaction between stromal and AML cells, see Rashidi et al. [85].

#### 4.1.3. MSC in Multiple Myeloma (MM)

Stromal cells isolated from MM patients display lower proliferative capacity, a premature senescence profile, and reduced ability to differentiate in osteoblasts compared to healthy MSC [61,86,87,88,89,90,91,92,93]. These features are usually associated with higher secretion of pro-angiogenic factors and modulated ability to impair T cell proliferation compared to healthy donors [58,66,94,95,96,97,98]. The BMSC/MM cell interaction involves adhesion molecules and soluble factors, which promotes both MM and stromal survival, proliferation and drug resistance [69]. Whereas soluble factors as IL-6, TGF-β, and TNF-α are well known to improve tumor and stromal survival, recent findings have shown that chemokines secreted by MM cells, such as CCL25, also play an important role in the recruitment of stromal cells [99] (Figure 2). In turn, MSC decrease the Bortezomib-induced apoptosis in MM cells (mainly by increasing cell cycle proteins and decreasing caspase-3 activation), facilitating tumor survival. More recently, it has been shown that MSC and plasma cells communicate with each other by secreting microvesicles [100] and microRNA [101], which promote MM cell growth in vitro. Among other adhesion molecules, CXCR4 plays an important role in MM/stromal cell interaction. Downregulated by the CXCL12 secreted by stromal cells, CXCR4 promotes MM cells’ migration and homing in the bone marrow [102]. Notably, stromal cells isolated from MM patients induce PD-L1 expression in MM cells, which promotes the drug resistance and immune escape of MM cells [103,104].

### 4.2. MSC and Cross-Talk with Tumor Cells in Carcinomas

Within solid tumors, MSC can produce and secrete several growth factors such as hepatocyte growth factor (HGF), insulin-like growth factor (IGF1), and fibroblast growth factor (FGF), which can directly influence the tumor cell growth [2,3]. In addition, MSC release TGF–β, which can display both tumor promoter or tumor suppressor functions, depending on the type of tumor [105]. TGF-β is a key factor in inducing and regulating the EMT of solid tumor cells [106]. Also, MSC can influence solid tumor fate, secreting vascular endothelial cell growth factor (VEGF) and platelet-derived growth factor (PDGF), which are involved in the neovascularization processes typical of the tumor niche [2,3]. In this context, the possibility of blocking tumor cell growth by inhibiting VEGF and/or the PDGF signaling axis is well established [107,108,109]. On the other hand, tumor cells can produce some of the same cytokines, angiogenic, and growth factors released by MSC such as IL-8, PDGF, EGF, TGF-β, IL-1β, and TNF-α, which can trigger MSC migration [110,111]. Several of the molecular mechanisms and biochemical pathways involved in MSC–solid tumor cross-talk have been extensively reviewed recently [112]. Here, we should point out that in these processes the exosomes released by MSC play a key role [113,114]. For instance, MSC-derived exosomes can influence the fate of nasopharyngeal carcinoma by modifying the expression of EMT markers [115]. In addition, the mechanisms of communication between MSC and tumor cells are well reviewed by Melzer and colleagues [116]. These findings strongly suggest that MSC-derived factors can play a key role in the regulation of solid tumor cell growth, apoptosis, and spreading.

## 5. MSC and Tumor Cells: The Cross-Talk which Impairs Innate Cell-Mediated Surveillance

**Figure 3:**
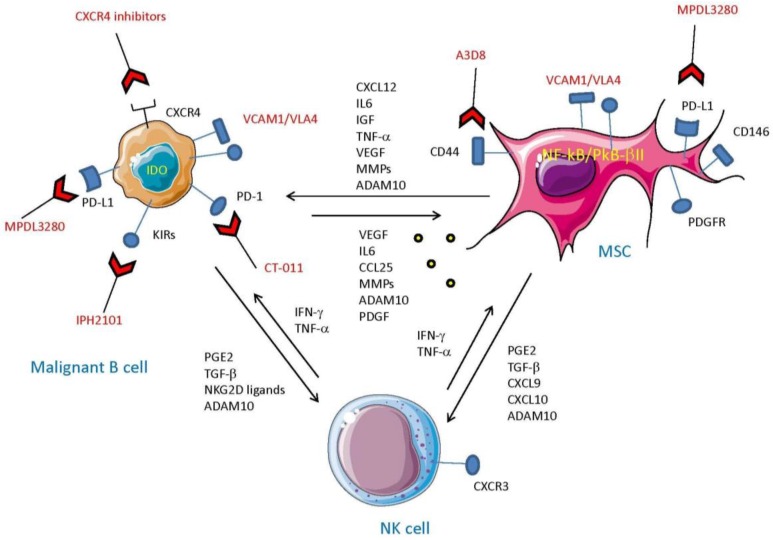
Interaction between mesenchymal stromal cells and malignant B cells to impair NK cell functions. The cross-talk between NK cells, MSC, and malignant B cells is complicated. NK cells producing IFN-γ and TNF-α can trigger both MSC and malignant B cells to produce inhibiting factors such as PGE_2_ and TGF-β that in turn downregulate NK cell-mediated anti-tumor immunity. Furthermore, the complex interplay of cytokines and growth factors between MSC and B cells can promote the survival and proliferation of both cell types. The increase in TME of matrix metalloproteases (MMP) and ADAMs, also present in exosomes, can favor a strong release of soluble ligands for activating receptors of effector cells (e.g., NKG2D-L), leading to a more robust impairment of NK cell activation. The different molecular structures that can be targeted on B and MSC are shown for completeness. Indeed, anti-PD-1 and PD-L1 antibodies (CT-011 and MPDL3280), as well as CXCR4 inhibitors, target relevant surface molecules whose engagement can break the established loop between MSC and B cells, mitigating the inhibition of NK cells. It should be noted that leukemic cell killing can also be restored using anti-KIR antibodies (IPH2101), thereby impairing the interaction between KIR expressed on NK cells and HLA on tumor cells and blocking the KIR-mediated negative signals delivered to NK cells.

Advanced findings support the fact that stromal and tumor cells interact with each other not only to promote their own survival, drug resistance, and proliferation, but also to escape NK cell surveillance [2,57,61] (Figure 3)*.* Vasold and colleagues have recently reported that AML cells cultured with stromal cells displayed a strongly reduced susceptibility to NK cell-mediated killing [117]. Additionally, this stromal-induced protection was cell-cell contact-dependent. More recently, it has been shown that BMSC secrete several chemokines to impair NK cell recognition in MM patients, thus promoting tumor growth and escape [97]. Interestingly, it has been shown that the secretion of CXCL9 and CXCL10 by stromal cells, associated with a downregulation of CXCL12 secretion, decreases CXCR3 expression in NK cells isolated from MM patients, acting as an “exit signal driving NK cells outside the bone marrow” [97]. On the other hand, it has been demonstrated that the CXCL12 secreted by stromal cells isolated from MM patients, acting via CXCR4, plays a critical role inretaining immature and mature NK cells in the BM [91,118]. These results demonstrate that the interaction between NK cells and the tumor microenvironment in MM patient needs further investigation.

Interestingly, contrasting with the idea that the stromal microenvironment protects tumor cells from NK cell attack, it has recently been shown that BMSC isolated from low-risk ALL patients promotes NK cell anti-tumor ability compared to healthy donors [119]. Indeed, ALL-derived stromal cells not only did not decrease activating receptors expression on NK cells, but also upregulated cytokine secretion, granule exocytosis, and cytotoxic functions. Interestingly, this could occur since ALL-isolated stromal cells express co-stimulatory molecules such as CD40 and CD86, normally not expressed in healthy donors [55,119]. Moreover, stromal cells isolated from ALL patients display low/negative expression of PD-L1, which is expressed on other hematological malignancies-derived stromal cells. It is also conceivable that the TGF-β produced during reciprocal cross-talk of MSC and solid tumor cells can affect the immune response of NK and any kind of T cell subset [120,121,122,123,124]. Indeed, TGF-β can downregulate the expression of the NKG2D-activating receptor on NK and γδ T cells, leading to impairment of tumor cells bearing NKG2DL [2,24,25,26]. In addition, microvesicles in hypoxic conditions derived from tumor cells and/or MSC can trigger additional mechanisms to suppress anti-tumor immunity [125]. Thus, the mutual cross-talk between MSC and tumor cells can strongly impair the innate immune response.

## 6. Drugs that Can Influence MSC-Mediated Immune Regulation

**Figure 4:**
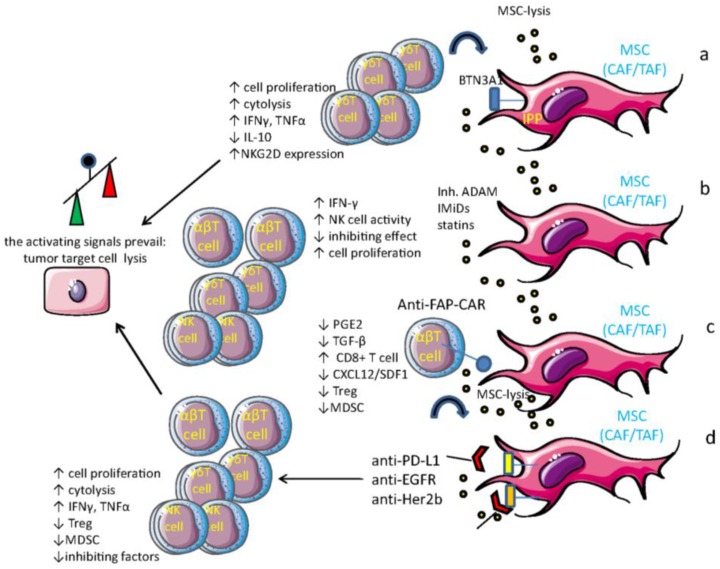
MSC as a target of tumor therapy. Several means can be used to relieve the MSC-mediated inhibition of anti-tumor immune response: (**a**) triggering Vδ2Vγ9 γδ T cells with aminobisphosphonates (N-BPs) such as zoledronic acid and accumulation in MSC of isopentenyl-pyrophosphate (IPP), which is presented by butyrophilin 3A1. This leads to (i) γδ T cell expansion; (ii) MSC elimination; and (iii) enhancement of anti-tumor cytolysis; (**b**) administration of ADAM inhibitors, immunomodulatory drugs (IMiDs), or statin, which impairs MSC’s assistance to tumor cells and triggers, at least for IMiDs, an immune reaction (ADAM inhibitors impair the shedding of NKG2D-L, increasing the NK cell-mediated recognition of target cells); (**c**) using T cells armed with chimeric anti-fibroblast activation protein (FAP)-antigen receptor (CAR) to eliminate MSC; (**d**) an analogous effect can be obtained with anti-EGFR and anti-Her2b humanized antibodies, which can activate antibody-dependent cytotoxicity (ADCC) and impair EGF-mediated pro-survival and proliferation effects. Overall, these different approaches can inhibit the establishment of a downregulating tumor microenvironment, increasing the immune reaction to tumor cells.

Several approaches can be used to target MSC to impair their influence in determining a TME prone to inhibit immune response: (a) drugs that inhibit MSC functions involved either in immune regulation or in favoring tumor cell growth; (b) drugs that specifically kill MSC present in TME; and (c) drugs that can revert their immunosuppressive properties switching the TME from immunoregulatory to immunostimulating (Figure 4). These drugs can impair MSC function, but they are not specific for tumor MSC as it happens for chemotherapeutic drugs. We have shown that hydroxy-methyl-glutaryl-coenzyme A (HMG-CoA) reductase inhibitors, also known as statins (which affect mevalonate and cholesterol synthesis), strongly inhibit the immunosuppressive functions of MSC [126,127,128,129,130]. This would suggest that cholesterol can be a suitable target of MSC. However, cholesterol is also a key molecule for immune effector cells such as NK or T cell lymphocytes [126,128]. NK cells incubated with statins cannot hit tumor cells through perforin and granzyme pathways [128], even if FasL- and TNF-α-mediated killing is still conserved [128]. Thus, to use statins to target MSC one should identify a molecular target more specific for MSC. In this context, it is conceivable that surface markers expressed mainly, if not exclusively, by MSC should be considered to regulate the TME. Indeed, FAP, CD73, and CD105 can be considered as suitable markers to target MSC [11,13,14,131,132,133,134,135,136,137,138,139,140,141,142,143,144,145,146,147,148,149,150]. In hematological malignancies, but also in solid tumors, the administration of immunomodulatory drugs (IMiDs) derived from their prototype Thalidomide to CC-122 has given very attractive results because these compounds can hit MSC besides tumor cells [151,152,153]. Among drugs that can induce killing of MSC, one should consider humanized monoclonal antibodies (huAb), which have mainly been used for the treatment of solid tumors because they are directed to receptors involved in their proliferation [154,155,156]. Indeed, MSC as CAF can express detectable levels of members of the EGFR family as such EGFR and Her2b. Thus, it is conceivable that administration of huAb to EGFR as cetuximab or to Her2b as trastuzumab can indeed target both tumor cells and MSC; further, either anti-PD-L1 or PD-L2 huAb can react with MSC, possibly triggering ADCC by FcγRIIIA^+^ NK and γδ T cells [2,3]. In addition, theanti-PD-1 and anti-PD-L1 huAb can relieve effector lymphocyte of one relevant immune-check point [157,158] and can also target MSC. Recently, we have shown that lymph node MSC derived from non-Hodgkin lymphoma patients primed with zoledronic acid can trigger, rather than inhibit, Vδ2 T cell proliferation [26]. Indeed, zoledronate-pulsed MSC reduce the secretion of the immunoregulatory cytokine TGF-β and increase IL-15 expression. This disequilibrium between TGF-β and IL-15 leads to effector γδ T cells lysing tumor lymphoma cells more efficiently [26]. As TGF-β plays a role in EMT [17,18,105,120,121,122,123,124], one can suggest that zoledronate can influence also EMT of tumor cell impairing tumor cell expansion and metastasization. Notably, zoledronate is commonly used for osteoporosis in post-menopausal women, thus it would be a suitable therapeutic tool in triggering the immune response in cancer and influencing MSC behavior. More importantly, selective inhibitors of ADAMs, which block the secretion of NKG2D-L from carcinomas, leukemic cells, and MSC, could be used to enhance tumor cell recognition [35,159]. These findings would suggest that MSC display several molecular targets suitable for therapy but no one of them is exclusively expressed by this cell population.

## 7. MSC as Target Cells for Anti-Tumor Vaccines

To boost host immune response against tumor cells, the possibility of administering anti-tumor vaccines is very attractive. Several kinds, compositions, and modes of administration of anti-tumor vaccines have been used with different results [160,161,162,163,164,165,166,167]. More recently, the focus of anti-tumor vaccines has been moved from tumor cells to TME too [7,9,10,131,132,133,134,135,136,137,138,139,140,141,142,143,144,145,146,147,148,149,150,151,152,153,154,155,156,157,158,159,160,161,162,163,164,165,166,167,168,169,170,171,172,173,174,175]. Indeed, the possibility of targeting tumor endothelial cells or the VEGF signaling axis with specific vaccines has been assessed in preclinical studies, and clinical trials are ongoing [168]. Most importantly, there are some reports that have indicated MSC as an attractive target for anti-tumor vaccines [12,133]. MSC such as CAF or TAF are the main source of collagen type I, which can interfere with the uptake of anti-tumor drugs [10,145]. Thus, targeting MSC can limit collagen type I production, thereby enhancing the sensitivity of tumor cells to chemotherapy. Recently, it has been shown in a murine system that targeting CAF and tumor cells is very effective in modifying TME. Indeed, tumor cells expressing FAP can be used as a vaccine, leading to elimination of solid tumors and their vascular dissemination. Importantly, this effect was characterized by the tumor infiltration of CD8^+^ T cells, reduction of intratumor CAF, and inhibition of recruitment of immunosuppressive cells within the tumor [148]. Interestingly, the FAP expressed on MSC has also been considered as a target for chimeric antigen receptor (CAR) T cells [137,146,147] or redirected T cells [169] (Figure 4). Indeed, a retroviral FAP specific construct has been developed composed of a single-chain Fv FAP, CD8α hinge, and transmembrane regions with the human CD3ζ and 4-1BB activation domains. Adoptively transferred mouse T cells transduced with this FAP-CAR construct inhibited the growth of several types of transplanted tumors through an increase in the CD8-mediated anti-tumor response. Furthermore, it has been reported that the administration of Tranilast, an anti-fibrotic agent, in murine models using E-G7 lymphoma, LLC1 Lewis lung cancer, or B16F1 melanoma cells resulted in an evident decrease of Treg and myeloid-derived suppressor cells (MDSC), associated with a decrease of stromal cell-derived factor-1 (SDF-1), PGE_2_, and TGF-β. Also in this case, an increment of CD8^+^ T cells specific to TAA, NK activity, and humoral immunity in combination with dendritic cell-based vaccines has been observed. It is of note that the inhibition of tumor growth was not evident in severe combined immunodeficient (SCID) mice, suggesting that a full immune response is needed to get efficient anti-CAF therapy [147].

## 8. Conclusions

In the last decade the treatment of different neoplasia with targeted molecular therapies has become a standardized and very efficient approach [175,176,177,178,179,180]. It is thought that these therapies hit tumor cells mainly by focusing on an essential biochemical pathway such as phosphokinases inhibitors, or on a tumor cell-relevant growth receptor with huAb. Usually, kinase inhibitors block cell proliferation, while monoclonal antibodies compete with soluble growth factor or trigger complementary and cell-dependent tumor cytotoxicity [175,176,177,178,179,180]. Furthermore, the administration of huAb, which relieves the strain to the host immune system, is increasing, leading to unexpected therapeutic results in highly resistant tumor cells [176,177,178,179,180]. In these latter instances, the molecules mainly targeted are the cytotoxic T-lymphocyte antigen 4 (CTLA-4) and programmed cell death 1 (PD-1) or its respective ligand, PD-L1 [176,177,178,179,180]. This enhances the immune reaction to neoplastic cells, supporting the notion that host lymphocytes can indeed efficiently recognize and eliminate tumor cells. The relevance in TME of fibroblast-like stromal cells, a heterogeneous cellular population comprising various kinds of mesenchymal cells involved in the regulation of anti-tumor immune response, is becoming evident. Evidence strongly suggests that these cells can be a suitable target to increase the anti-tumor immune response; indeed, it is possible to use drugs, vaccines, and CARs to try and boost their immunosuppressive properties. The identification of a peculiar MSC marker can aid with specifically targeting the stroma and influencing TME-impairing tumor cell growth and spread.

## Abbreviations

ADAM	a disintegrin and metalloprotease
ADCC	antibody-dependent cellular cytotoxicity
ALL	acute lymphoblastic leukemia
AML	acute myeloid leukemia
AMP	adenosine monophosphate
BM	bone marrow
BMSC	bone marrow stromal cells
CAFs	carcinoma-associated fibroblasts
CAR	chimeric antigen receptor
CDC	complement-dependent cytotoxicity
CLIR	C-lectin type inhibitory receptors
CLL	chronic lymphocytic leukemia
CML	chronic myelogenous leukemia
CTL	cytolytic T lymphocytes
CTLA-4	cytotoxic T lymphocyte antigen 4
DC	dendritic cell
DNAM-1	DNAX accessory molecule 1
ECM	extracellular matrix component
EMT	epithelial–mesenchymal transition
EGF	epidermal growth factor
EGFR	epidermal growth factor receptor
FAP	fibroblast activation protein
PGE_2_	prostaglandin E_2_
HSC	hematopoietic stem cells
Her2b	epidermal growth receptor 2b
HGF	hepatocyte growth factor
HL	Hodgkin lymphoma
HLA	human class I leukocyte antigen
KIR	killer Ig-like inhibitory receptor
IDO	indoleamine 2,3, deoxigenase
IFNγ	interferon γ
IL	inteleukin-2, 6, 10, 12, 15, 18
LILRB	leukocyte immunoglobulin-like receptors
LNMSC	lymph node MSC
MDSC	myeloid-derived suppressor cells
MICA/B	MHC class I polypeptide related sequence A/B
MM	multiple myeloma
MMP	membrane metalloprotease
MSC	mesenchymal stromal cells
NCR	natural cytotoxicity receptor
NHL	non-Hodgkin lymphoma
NK cell	natural killer cell
NKG2D	natural-killer group 2 member D
NKG2DL	NKG2D ligand
NKT	natural killer-like T cells
N-BPs	aminobisphosphonates
PD1	programmed death-1
PDL1	programmed death ligand-1
P4H	prolyl-4-idroxilase
SCID	severe combined immunodeficiency
SDF-1	stromal-derived factor 1
SMA	smooth muscle actin
TAA	tumor-associated antigen
TACE	tumor necrosis factor-α-converting enzyme
TAF	tumor-associated fibroblasts
TIGIT	T cell immunoglobulin and ITIM domain
TGFβ	transforming growth factor β
TNFα	tumor necrosis factor α
Treg	regulatory T cells
ULBP1-6	UL16 binding protein 1–6
